# A comparison between the pathophysiology of multiple sclerosis and normal pressure hydrocephalus: is pulse wave encephalopathy a component of MS?

**DOI:** 10.1186/s12987-016-0041-2

**Published:** 2016-09-22

**Authors:** Grant A. Bateman, Jeannette Lechner-Scott, Rodney A. Lea

**Affiliations:** 1 Department of Medical Imaging, John Hunter Hospital, Locked Bag 1, Newcastle Region Mail Center, Newcastle, 2310 Australia; 2 Newcastle University Faculty of Health, Callaghan Campus Newcastle, Newcastle, Australia; 3 Department of Neurology, John Hunter Hospital, Newcastle, Australia; 4 Hunter Medical Research Institute, Newcastle, Australia; 5 Institute of Health and Biomedical Innovation, Queensland University of Technology, Brisbane, Australia

**Keywords:** Multiple sclerosis, Normal pressure hydrocephalus, Cerebral blood flow, Pulse wave encephalopathy, Compliance, Cerebrospinal fluid

## Abstract

**Background:**

It has been suggested there is a chronic neurodegenerative disorder, underlying the pathophysiology of multiple sclerosis (MS), which is distinct from the more obvious immune-mediated attack on the white matter. Limited data exists indicating there is an alteration in pulse wave propagation within the craniospinal cavity in MS, similar to the findings in normal pressure hydrocephalus (NPH). It is hypothesized MS may harbor pulse wave encephalopathy. The purpose of this study is to compare blood flow and pulse wave measurements in MS patients with a cohort of NPH patients and control subjects, to test this hypothesis.

**Methods:**

Twenty patients with MS underwent magnetic resonance (MR) flow quantification techniques. Mean blood flow and stroke volume were measured in the arterial inflow and venous out flow from the sagittal (SSS) and straight sinus (ST). The arteriovenous delay (AVD) was defined. The results were compared with both age-matched controls and NPH patients.

**Results:**

In MS there was a 35 % reduction in arteriovenous delay and a 5 % reduction in the percentage of the arterial inflow returning via the sagittal sinus compared to age matched controls. There was an alteration in pulse wave propagation, with a 26 % increase in arterial stroke volume but 30 % reduction in SSS and ST stroke volume. The AVD and blood flow changes were in the same direction to those of NPH patients.

**Conclusions:**

There are blood flow and pulsation propagation changes in MS patients which are similar to those of NPH patients. The findings would be consistent with an underlying pulse wave encephalopathy component in MS.

**Electronic supplementary material:**

The online version of this article (doi:10.1186/s12987-016-0041-2) contains supplementary material, which is available to authorized users.

## Background

Multiple sclerosis (MS) is considered to be an autoimmune inflammatory demyelinating disease of the central nervous system. The histopathology of MS shows inflammation to be most prominent in the white matter of the corpus callosum, subcortical white matter tracts, optic nerve and spinal tracts. There is T cell and macrophage-mediated damage leading to demyelination [1]. The immune-mediated aspects of MS dominate the research literature with all of the currently available therapies directed at modulating the immune response in this disease [2]. Despite the overwhelming dominance of the immune hypothesis in MS research, in a review of the literature, Juurlink noted that although the immune-modulating therapies reduce the number and severity of exacerbations, they do not prevent the long-term progression toward disability in MS [3]. If the amount of brain inflammation occurring during the disease process does not affect the degeneration, this suggests there may be some neurodegenerative process, other than the inflammation, mediating the slow decline. Although the autoimmune response of the body could secondarily elicit a neurodegenerative effect, in a major review Stys suggests that it was also possible that MS may be a degenerative disease that secondarily elicits an autoimmune response [4]. In the second scenario, the neurodegenerative disorder would perhaps lead to a breakdown of the blood–brain barrier and damage the myelin, thus leaving the myelin susceptible to immune attack in those individuals who were prone to such a response. Indeed, given that multiple sclerosis is characterized by simultaneous focal breakdown of the blood–brain barrier, the development of demyelinating lesions that are associated with immune-mediated inflammation and eventual axonal loss [3], it may be hard to discern which is the initiating event and which the secondary.

Multiple sclerosis has been noted to have some characteristics in common with normal pressure hydrocephalus (NPH). On the basis of decreased intracranial compliance [5], increased ventricular [6] and Virchow–Robin space size [7], the presence of Hakim’s triad [8–10], increased pulsatility of the CSF through the aqueduct [11], reduced net flow of CSF through the same structure [12] together with decreased CSF pulsation at C2 level [13] (all of which are noted in NPH [3]), Juurlink suggested the possible initiating neurodegenerative process could be an underlying pulse wave encephalopathy [3]. Pulse wave encephalopathy was a concept originally developed by one of the current authors to describe how three of the causes of dementia (Alzheimer’s disease, vascular dementia and normal pressure hydrocephalus) could be interrelated along a common pathophysiological spectrum [14]. The spectrum was suggested to be related to the strength of the pulse pressure waves induced in the craniospinal cavity by the arterial tree and the way the pulse waves interact with the compliance of the spinal canal and venous system [14]. It was hypothesized, increased arterial pulse pressure, if not damped sufficiently (either because it was too large or the available compliance of the spinal canal and/or veins too small), would lead to elevated capillary and venous pulse pressure and increased aqueduct pulsation (together with other pathological effects) [14]. However, the physiological evidence Juurlink has used to justify MS as being similar to NPH is tenuous at best. Firstly, Gorucu commented that the increase in stroke volume through the aqueduct in MS could be caused by hyperdynamic dilated ventricles secondary to the underlying atrophy [11]. Therefore, the increased aqueduct flow could be passive, rather than part of an active, pulsation driven process. Secondly, the estimation of net CSF flow through the aqueduct using MRI is currently under a cloud. Due to the very low flow rates being measured, the cumulative error in net aqueduct flow measurement is estimated to be up to 30 % [15]. Therefore, this may not be a valid metric. Finally, the reduced stroke volume through the spinal canal in MS was dismissed by the authors of the paper where it was originally described because there was also a reduction in the arterial blood flow in the MS patients. It was assumed there would have been an associated reduced arterial stroke volume and therefore less impetus to move the spinal canal CSF [13]. Therefore, much of the data is missing from the literature which would enable one to answer the question, “Is there an underlying pulse wave encephalopathy component in MS?” The required evidence includes an accurate measure of the total arterial pulsation stroke volume (which is the input into the system driven by pulse pressure), the intracranial compliance (which is the moderator of pulse pressure) and the stroke volume of the venous system (which is an output of the system). The purpose of the current study is to measure these variables utilizing a phase contrast MRI technique directed to the arterial input and venous output in a cohort of MS patients and compare them with normal controls and NPH patients.

## Methods

### Subjects

Patients with multiple sclerosis (MS) are regularly followed-up with routine MRI scans. As part of the standard protocol, MR flow quantification sequences were added to provide a surrogate marker of cerebral metabolism and brain volume. In the current study, twenty consecutive relapsing remitting MS patients had these sequences reviewed, all were female. The demographic details are summarized in Table 1. The mean age was 43 ± 12 year. The mean length of time from first symptoms to the MRI was 13 ± 8 year. The mean audio recorded cognitive screen score was 85 ± 12. The mean extended disability status scale for the patients was 2.5 ± 1.8. Seventeen patients were currently undergoing immunomodulation therapy at the time of the study; the other three had not received treatment for over a year. Twenty patients with NPH, who were previously studied with the same flow quantification technique (with the results being previously published in part [16]), were selected for comparison. This group comprised 12 males and 8 females. The NPH group was originally selected on the basis of the classical clinical triad of gait ataxia ± dementia ± incontinence together with ventriculomegaly. All 20 patients were confirmed to have NPH, having shown clinical improvement following CSF flow diversion (see [16] for more details). The controls were selected from a bank of spouses and volunteers recruited by advertisement and have been previously published [16, 17]. The controls were confirmed to be without structural brain pathology on standard MRI and underwent Mini-Mental State examination and formal neuropsychological testing. The normal young were selected to match the MS patients and averaged 43 ± 16 year, all were females. The normal elderly averaged 74 ± 16 year and there were 6 women and 6 men.

**Table 1:** Demographics and clinical information for multiple sclerosis patients

Patient number	Age (years)	Sex	Duration of disease (years)	Type	Treatment	ARCS	EDSS
1	21	F	9	RRMS	Natalizumab	79	1.5
2	38	F	2	RRMS	Interferon beta-1a	100	0
3	57	F	4	RRMS	Betaferon	73	1.5
4	36	F	11	RRMS	Natalizumab	78	5.5
5	46	F	21	RRMS	Natalizumab	81	6.0
6	62	F	17	RRMS	Nil	72	6.5
7	40	F	16	RRMS	Natalizumab	82	1.5
8	51	F	26	RRMS	Nil	90	2.5
9	40	F	13	RRMS	Natalizumab	77	3.5
10	31	F	9	RRMS	Natalizumab	71	3.0
11	50	F	28	RRMS	Nil	96	2.5
12	36	F	4	RRMS	Natalizumab	83	1.0
13	46	F	4	RRMS	Fingolimod	86	1.5
14	59	F	22	RRMS	Fingolimod	60	1.5
15	36	F	4	RRMS	Interferon beta-1a	95	1.5
16	62	F	25	RRMS	Glatiramer	93	1.5
17	47	F	14	RRMS	Betaferon	92	1.0
18	31	F	9	RRMS	Natalizumab	79	1.5
19	28	F	11	RRMS	Natalizumab	93	1.5
20	48	F	5	RRMS	Immunoglobulin	111	3.5

*ARCS* audio recorded cognitive screen; *EDSS* extended disability status scale; *F* female; *RRMS*,relapsing remitting multiple sclerosis

### MR and analysis

**Fig. 1:**
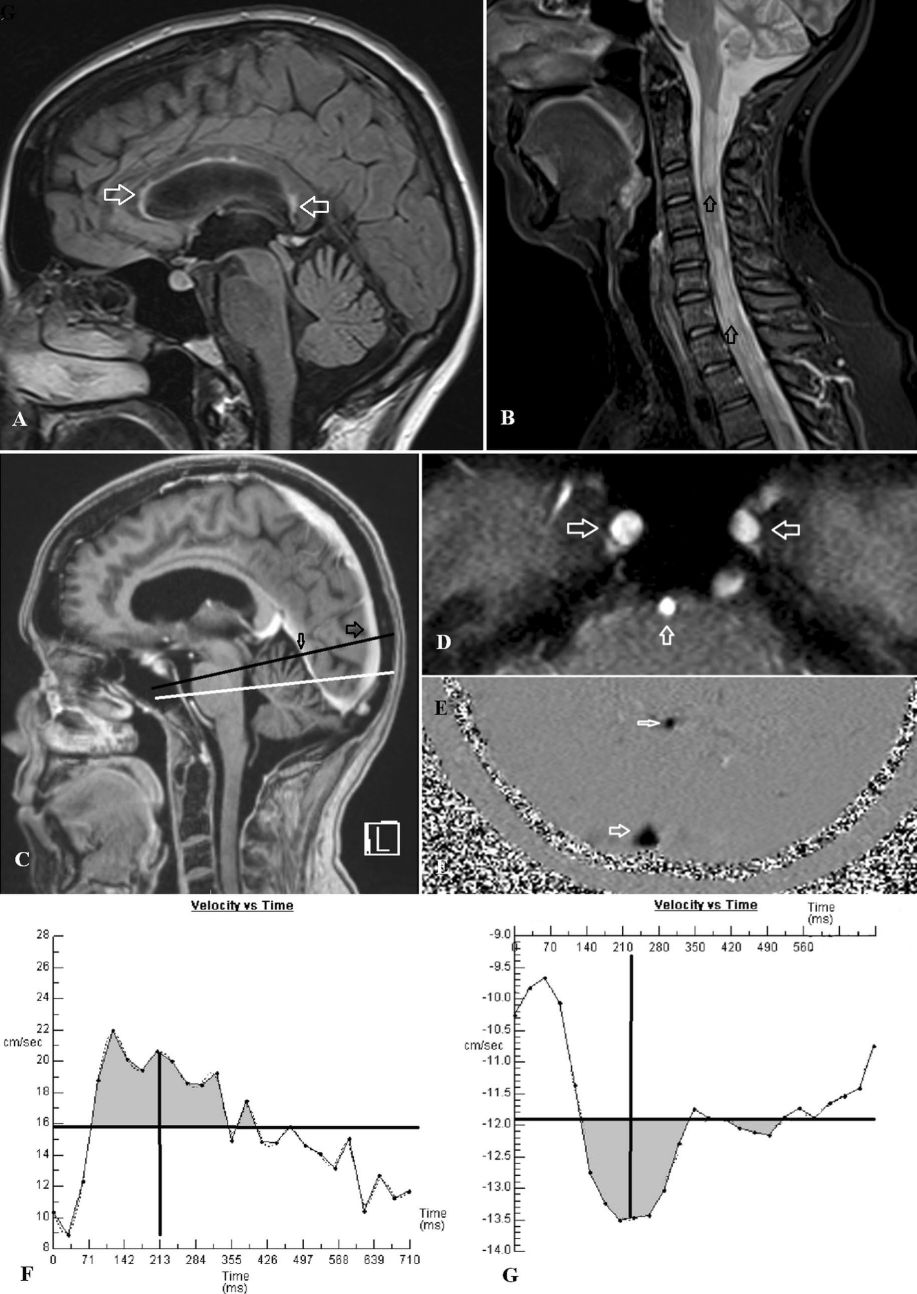
**A** A FLAIR sagittal image of a 48 year old female MS patient with *arrows* showing some plaques within the corpus callosum but no significant cerebral volume loss. **B** A T2 sagittal image of the cervical spine in the same patient with arrows showing extensive MS plaques. **C** A sagittal post contrast T1 image with the *black line* showing the position of the venous acquisition passing through the sagittal sinus (*large arrow*) and straight sinus (*small arrow*). The *white line* is the arterial acquisition passing through the skull base. **D** The localizer image from the arterial acquisition showing the carotid arteries just above foramen lacerum (*horizontal arrows*) and the mid basilar artery (*vertical arrow*). **E** The phase image from the venous acquisition showing the sagittal sinus (*large arrow*) and straight sinus (*small arrow*). **F** The arterial flow graph from the same patient. The *horizontal line* represents the mean flow velocity. Where the mean velocity transects the flow graph is the start and end of systole. The *grey area* above the *line* represents the arterial stroke volume which for the three arteries combined was 1184 µL. The *vertical line* represents the midpoint of the two transection points and is the midpoint of the primary arterial wave i.e. 213 mS. **G** The sagittal sinus flow graph from the same patient. The *horizontal line* is the mean flow velocity. The *grey area* is the sinus stroke volume which is 118 µL or 10 % of the arterial stroke volume. The *vertical line* is the midpoint of the two transection points and is the center of the primary pulse wave i.e. 223 mS giving an AVD of 10 mS in this patient which is less than 10 % of normal indicating very low compliance

All patients were imaged on a 1.5 T superconducting magnet (Vario; Siemens, Erlangen Germany). In the brain, the patients were scanned with standard T1 and FLAIR sagittal images followed by T2 axial and diffusion-weighted axial images. A 3D T1 post contrast series was acquired. In the neck, post contrast T1 sagittal, inversion recovery sagittal and T2 axial images were performed. Two MR phase contrast flow quantification sequences were acquired with retrospective cardiac gating. The TR was 26.5 ms, TE 6.9 ms, flip angle 15º, slice thickness 5 mm, matrix 192 × 512, FOV 150 and a single excitation. The velocity encoding value was 75/s for the arterial acquisition and 40 cm/s for the venous. The arterial plane was selected to pass through the vertical segments of the carotid and basilar arteries at the skull base. The venous plane was selected to pass through the sagittal sinus above the torcular and then pass through the mid part of the straight sinus (Fig. 1C–E). The planar imaging, as well as the flow quantification raw data, was archived on DVD discs.

Regions of interest were placed around the carotid arteries, basilar artery, the sagittal sinus and the straight sinus in each patient. Care was taken to exclude aliasing by retrospectively manipulating the base lines of each resultant graph giving an effective arterial flow limit of 150 cm/s. Background subtraction was utilized to limit the effect of eddy currents. The addition of the flow from the three arteries gave the total arterial inflow. The sagittal sinus and straight sinus outflow was obtained from the regions of interest placed around these vessels. The percentage of the arterial inflow drained by each sinus was calculated for each patient.

The arteriovenous delay (AVD) is the time taken between the center of the primary arterial pulse and the center of the primary sagittal sinus outflow pulse. The arterial and venous waveforms were transected by a horizontal line representing the mean blood flow for each graph. The midpoint between the two sites where the mean flow crosses the flow curve is taken to be the midpoint of the pulse wave. Subtracting the arterial and sagittal venous midpoints gave the AVD. This is inversely proportional to the pulse wave velocity between these two points (Fig. 1F, G). The arterial pulse volume represents the degree to which the arterial tree expands in systole and is calculated from the graphs obtained from each carotid and basilar artery. The mean blood flow velocity for each artery for the entire heartbeat was subtracted from the mean blood flow velocity for the period of systole (systole was defined as the section of the graph between the transection points) for the same artery, giving the mean increase in flow velocity over systole for that vessel (Fig. 1F). This value when multiplied by the time taken for systole (the distance between transection points) to occur and the cross-sectional area of the vessel (region of interest) gives the volume of expansion of that vessel in systole. The addition of the value of vessel expansion obtained for both carotid and the basilar arteries gave the arterial stroke volume. The sagittal and straight sinus stroke volumes were obtained for each patient by a similar process to the arterial stroke volume except using the venous flow data (Fig. 1G). The percentage of the arterial stroke volume represented by each venous stroke volume was calculated.

### Statistical analysis

Group means and standard deviations were obtained for each of the measurements. Comparison of mean differences between the study groups were tested using a general linear model–univariate analysis of variance (ANOVA). For each ANOVA, the measurement was entered as the dependent variable, with study group entered as a fixed factor and age entered as a covariate between study groups and relevant controls. All measurements were checked for normality using the one-sample Komogorov–Smirnov test and for equality of variance using the Levene’s Test. A non-paired T test, with a *p* value of less than 0.05 was used to indicate statistical significance. All statistical analyses were performed using IBM^®^ SPSS Statistics V22.

## Results

The raw data supporting the findings of this study can be found in Additional file 1. The blood flow data is summarized in Table 2 with the compliance and pulsation data summarized in Table 3. The arterial inflow, SSS outflow, AVD and arterial stroke volume data for the normal elderly and NPH patients has been previously published [17]. The ST outflow and both venous sinus stroke volumes were not previously published and were calculated from the original raw data retrieved from the archive DVD discs. The MS and normal young data has not been previously published.

**Table 2:** Brain blood flow in normal, multiple sclerosis and normal pressure hydrocephalus patients

	Age (years)	Arterial inflow (ml/min)	SSS outflow (ml/min)	ST outflow (ml/min)	%SSS	%ST
Normal young n = 20						
Mean	43	792	356	108	46	14
SD	16	142	76	29	8	3
Normal elderly n = 12						
Mean	70	709	310	91	44	13
SD	5	110	65	30	9	4
*p* value NY vs NE	0.0001*	NS	0.03*	NS	NS	NS
Multiple sclerosis n = 20						
Mean	43	783	311	95	41	12
SD	12	160	44	28	7	4
*p* value MS vs NY	NS	NS	0.01*	NS	0.05*	NS
Normal pressure hydrocephalus n = 20						
Mean	74	567	197	68	36	13
SD	7	148	49	24	9	5
*p* value NPH vs NE	NS	0.02*	0.0005*	0.05*	0.03*	NS

*SSS* sagittal sinus sinus; *ST* straight sinus;  *%SSS* percentage of arterial inflow drained by the SSS;  *%ST* percentage of arterial inflow drained by ST; *SD* standard deviation; *t test *p* value <0.05; *MS* multiple sclerosis; *NS* not significant; *NPH* normal pressure hydrocephalus; *NY* normal young; *NE* normal elderly; *p* values are adjusted for age

**Table 3:** AVD and pulsation stroke volume

	AVD (ms)	Arterial SV (µL)	SSS SV (µL)	ST SV (µL)	%SSS SV	%ST SV
Normal young n = 20						
Mean	112	914	273	73	31	8.7
SD	67	264	96	30	9	3.9
Normal elderly n = 12						
Mean	111	1331	308	71	23	5.6
SD	50	374	124	20	7	1.8
*p* value NY vs NE	NS	0.0006*	NS	NS	0.02*	0.02*
Multiple sclerosis n = 20						
Mean	73	1152	188	50	16	4.4
SD	39	350	44	30	9	3.0
*p* value MS vs NY	0.02*	0.02*	0.007*	0.02*	0.0001*	0.0003*
Hydrocephalus n = 20						
Mean	47	963	170	42	19	4.4
SD	24	375	91	26	8	2.3
*p* value NPH vs NE	0.0002*	0.02*	0.0005*	0.002*	NS	NS

*AVD* arteriovenous delay; *ms* milliseconds; *SV* stroke volume; *SSS* superior sagittal sinus; *ST* straight sinus; *SD* standard deviation; *t test p value <0.05; *MS* multiple sclerosis; *NPH* normal pressure hydrocephalus; *NS* not significant; *NY* normal young; *NE* normal elderly; p values are adjusted for age

### Blood flow

As expected, there was a non-significant reduction in arterial inflow with normal aging between the two control groups, with the reduction in blood flow being approximately 3 ml/min/year. The sagittal sinus showed a reduced outflow with aging in proportion to the inflow. There was no significant alteration in the percentage of the blood flow returned by each sinus compared to the arterial inflow. The multiple sclerosis patients were compared to the normal young. There was no significant difference in arterial inflow. There was a 13 % reduction in sagittal sinus outflow (*p* = 0.01), which gave a 5 % reduction in the percentage of the arterial inflow drained by this sinus (*p* = 0.05). The straight sinus flow and percentage return were not significantly different to the controls. The NPH patients were compared to the normal elderly. The arterial inflow, SSS and ST outflow volumes were significantly reduced by 20, 36 and 25 % respectively (*p* = 0.02, *p* = 0.0005, *p* = 0.05). The percentage of the arterial inflow returned by the SSS was reduced by 8 % (*p* = 0.03) but the ST as a percentage of the inflow was not significantly different from normal.

### Craniospinal compliance and pulsation

There was no change in the AVD with aging. The arterial stroke volume was increased by 46 % in the normal elderly compared to the normal young (*p* = 0.0006). Despite the increase in arterial stroke volume, the two sinus stroke volumes were not significantly different between the control groups. However taken as a percentage of the arterial stroke volume both the venous stroke volumes were reduced with aging (*p* = 0.02 and *p* = 0.02 respectively). In MS the AVD was reduced by 35 % compared to the normal young (*p* = 0.02). There was an increase in arterial stroke volume of 26 % (*p* = 0.02) but a decrease in SSS and ST stroke volumes of 31 and 32 % respectively (*p* = 0.007 and *p* = 0.02). Thus the percentages of the arterial stroke volume directed to the venous sinuses were greatly reduced (*p* = 0.0001 and *p* = 0.0003 respectively). In NPH the AVD was reduced by 58 % (*p* = 0.0002) compared to the normal elderly. The arterial stroke volume, SSS and ST stroke volumes were reduced by 28, 45 and 41 % (*p* = 0.02 *p* = 0.0005 and *p* = 0.002 respectively), however, as a percentage of the arterial stroke volume the venous stroke volumes were not significantly altered.

## Discussion

There is a hydrodynamic spectrum underlying various forms of dementia. Vascular dementia is associated with increased arterial but normal venous stroke volume [17] with normal craniospinal compliance [18], Alzheimer’s disease is associated with normal arterial and venous stroke volume [17] but reduced craniospinal compliance [16] and normal pressure hydrocephalus is associated with reduced arterial and venous stroke volume and a very low craniospinal compliance. The multiple sclerosis patients in the current study show increased arterial but reduced venous stroke volume together with reduced craniospinal compliance. Therefore, MS overlaps the other three forms of dementia but is most closely correlated with NPH.

The pulse pressure of the blood flowing from the arteries into the arterioles and then into the capillaries is normally damped as it proceeds. The pulse induces a change in volume of blood in the arteries over the cardiac cycle. However, there is non-pulsatile continuous flow proceeding into the capillaries [17]. The mechanism required to bring about the change in pulsation is known as the windkessel effect [19]. Arterial damping depends on expansion of the arteries in systole and contraction in diastole. This removes some energy from the flow in systole and returns it in diastole. The Monro–Kellie doctrine states that as the skull is rigid and the CSF incompressible, the volume of the arterial pulse stored in systole must be accommodated by the available compliance i.e. either the walls of the container (the dura mater) must be shifted to allow egress of CSF from the cranial to spinal cavities and/or the veins passing through the subarachnoid space must be compressed [20]. Thus the arterial tree can only be as compliant as the walls of the container allow. It is envisaged a breakdown in the windkessel effect will direct greater pulse pressure into the capillary beds of the neural structures leading to parenchymal derangement [18]. A larger CSF pulse pressure will also be transmitted through the thin walled veins as they traverse the subarachnoid space and increase the venous pulse pressure. For example, Alzheimer’s disease (AD) is associated with a reduction in AVD of 36 % [16] which is almost identical to the MS patients in this study. The arterial pulse volume in AD is 1150 µL [17] which is also identical to MS. Is one neurodegenerative disease related to the other? It was suggested an increased capillary pulse pressure could account for the coiling and beading of the capillaries as well as the basement membrane disruption found in AD [18]. In the Framingham offspring study, higher central arterial pulse pressure was associated with lower brain volume, white matter hyperintensity and vascular and Alzheimer’s type cognitive aging [21]. In another study, measurements of the arterial pulse and the invasively-measured CSF pulse pressure were strongly associated with temporal lobe and hippocampal volume loss [22], suggesting pulse waves may damage the brain. Finally, Chandra suggests a common mechanism for the neurodegeneration found within MS and AD due to increased amyloid precursor protein expression in the axons around MS plaques [23]. The purpose of the current study was to measure the arterial pulsation, the available compliance and the outflow venous pulsation to determine if there is a breakdown in the windkessel effect in multiple sclerosis, similar to NPH.

### Blood flow changes

The arterial inflow to the brain reduces throughout life. In a cohort of normal controls of mean age 25 year previously studied by our group, the arterial inflow was 900 ml/min with the sagittal sinus returning 45 % of the arterial flow and the straight sinus 14 % [17]. In the current study, the normal young were mean age 43 year, the mean arterial inflow was 792 ml/min, the sagittal sinus returning 46 % and straight sinus 14 % of the flow. In the normal elderly of average age 70 year, the arterial inflow was 709 ml/min, the sagittal sinus returning 44 % and the straight sinus 13 % of the flow. Note the percentage of the arterial inflow returned by each sinus has not changed significantly over the 40 years spanned. The arterial inflow is noted to have reduced by 4.2 ml/min/year from 25 to 70 year which is similar to the findings of Stoquart–ElSankari et al. [24].

In normal pressure hydrocephalus there is a 23 % reduction in arterial inflow compared to the age matched controls. The reduction in sagittal sinus flow is somewhat larger at 36 % giving an 8 % reduction in the percentage of the inflow returning via this sinus. This is in comparison to the straight sinus where the percentage of the inflow is maintained. It has been previously noted in NPH that there is a 29 % reduction in sagittal sinus flow compared to controls with a 28 % increase in flow following CSF diversion [25]. Further investigation into this effect showed that the arterial inflow was unchanged. This indicates there must have been increased collateral flow bypassing the sinus before the shunt, which returns back to the normal venous pathway after the shunt. This suggests there is an elevation in sagittal sinus pressure but not straight sinus pressure in NPH which is reversible [26].

In MS, there was no significant difference in the arterial inflow between patients and controls. Some have suggested a reduction in total cerebral blood flow seen in some MS studies is due to small increases in venous pressure [27]. This would appear to be unlikely given the large perfusion pressure reserve available to the brain. However, increasing the sagittal sinus pressure by 1–2 mm Hg could affect the venous outflow by increasing collateral flow. Note that similar to NPH, the sagittal sinus returned 5 % less blood as a percentage compared to controls in MS. A previous study showed further evidence of increased SSS pressure in MS: strain-gauge plethysmography demonstrated a 63 % increase in the total venous resistance from the head in MS patients compared with healthy controls [28]. Given the pressure gradient from the sagittal sinus to the right heart is 2.5 mm Hg [29], this would give an increase in sinus pressure of 1.6 mm Hg if the blood flow was maintained. Indeed, direct measurement of the sinus pressure has shown an elevation of over 2 mm Hg in selected MS patients [30]. So it can be seen that there are similarities between the blood flow in NPH and MS patients.

### Arterial stroke volume

The arterial stroke volume is the expansion of the arterial tree in systole, over and above the mean flow and this is the impetus for both displacement of CSF into the spinal canal and compression of the cortical veins [31]. Normal aging showed an increase in the stroke volume between the two control groups of 46 %. It has been previously suggested that aging is associated with reduced compliance in the walls of the central arteries and that the larger pulse pressure waves generated by aging would penetrate deeper into the microcirculation, microvascular disease would ensue, with the brain and kidney being most susceptible [32]. Note that in the present study the arterial stroke volume increased with aging despite the reduction in non-pulsatile blood flow (refuting the previously discussed assertion they are always linked [13]). In MS there was a 26 % increase in arterial stroke volume compared to the age-matched controls. Fjeldstat et al [33] found patients with MS have lower central arterial compliance than healthy controls, which preferentially affects the CNS vessels. The pulse wave velocity between the brachial and ankle arteries is significantly higher in MS patients compared to controls, indicating stiffer central arteries [34]. Both these findings place a larger pulse pressure wave within the carotid vessels which must be damped. Higher central arterial pulse pressure is also associated with worsening gait performance in MS but not controls, suggesting altered vascular compliance may contribute to the deterioration in physical function in MS [35]. In comparison, NPH is associated with a reduced arterial stroke volume compared to controls, indicating a point of difference between NPH and MS.

### Arteriovenous delay

Compliance is defined as the ratio of the change in volume which occurs in a structure divided by the change in pressure which brings this about i.e. C = ΔV/ΔP [19]. Aortic compliance is estimated by measuring the time the pulse wave takes to travel from the brachial to the ankle arteries [36] because the pulse wave velocity is inversely proportional to compliance. Thus, craniospinal compliance can be measured invasively (1) by injecting a volume of fluid into the subarachnoid space and measuring the change in pressure which occurs, or non-invasively (2) by measuring the time the pulse wave takes to traverse the available space. The AVD measures between the arteries at the skull base and the venous sinuses. It could be assumed that the pulse traversed the capillary bed, passed along the cortical veins to directly enter the sinuses but this was not so. Direct measurement of the pulse wave timing has shown that the peak pulse in the cortical veins lags behind the sinuses indicating that the pulse volume exits the arterial tree passes into the subarachnoid space and re-enters the cortical vessels just before their junction with the sinuses [37]. Therefore the pulse volume passes into the spinal canal with a minimal time lag [38] and then travels to the venous outflow with a time lag measured by the AVD. The time taken for the pulse to travel from the arteries to the sagittal sinus is thus a measure of the compliance of the arteries, subarachnoid space and veins between these two places. It is undefined how this would directly correlate with the other invasive techniques used to measure craniospinal compliance. From the present study, we note that the AVD does not change from the normal young to the normal elderly similar to a previous study [24] suggesting the compliance of the craniospinal system remains unchanged with normal aging. This is in comparison with the findings in NPH, in which the present study shows a 58 % reduction in AVD compared to age matched controls. Previously, in a smaller cohort, the compliance was estimated to be reduced by 50 % in NPH [25], Mase et al. [39] using another MRI technique confirmed a 64 % reduction in craniospinal compliance in NPH. The compliance as measured by the AVD was reduced in MS by 35 % compared to age match controls, indicating another similarity between the patient groups being currently studied: i.e. both NPH and MS have reduced compliance.

In a previously published control group of average age 33 year, the mean CSF pulse pressure at C1/C2 was 1.6 ± 0.6 mm Hg [40]. In one study, it has been noted the CSF pulse pressure in NPH is twice normal [41] and 2–3 times normal in another study [42]. Finally, NPH patients who responded to shunt had intracranial pulse pressures averaging over 4 mm Hg [43]. Note, that despite the arterial stroke volume in NPH being 28 % less than age-matched controls in this study, the pulse pressure in the subarachnoid space has been shown to be twice normal [41, 42]. This indicates very low compliance as discussed above. In MS the arterial stroke volume was increased by 26 % and the compliance as measured by the AVD was reduced by 35 % compared to the normal young, suggesting the possibility of an increased pulse pressure in the subarachnoid space in MS. The literature provides some evidence for this, the estimated peak-to-peak pulse pressure gradient in the spinal canal in MS patients was noted to be doubled compared to controls [44] and the pulse volumes were significantly increased in the epidural veins in the spine in adolescents with MS [45] suggesting an increase in spinal pulse pressure.

### Local spinal canal compliance

Stivaros and Jackson noted, that displacement of CSF through the foramen magnum into the spinal subarachnoid space occurs due to the high compliance of the spinal arachnoid mater, accounting for at least 50 % of the pulse pressure volume displacement [46]. In a cohort of normal controls vs MS patients, the C2 stroke volume was reduced by 24 % [13]. The Monro–Kellie doctrine is essentially a restatement of the conservation of mass (if the arterial tree increases in volume then the CSF and venous volume must reduce by the same amount). If the spinal canal has the greater relative compliance than the veins then a larger percentage of the arterial stroke volume will be directed to the spine, if the veins are relatively more compliant than the spinal canal then their stroke volume will be greater as a percentage. In MS the arterial stroke volume was 26 % larger than controls but the percentage of the volume directed to the spinal canal was reduced by 24 % so we can conclude the spinal canal is much less compliant than normal in MS. One study found that there is no difference in the C2 stroke volume between normal elderly (at 71 year) and NPH [47]. In another study, the cervical stroke volume was reduced by 27 % compared to controls [48]. Given there is a 28 % reduction in arterial pulse volume in NPH compared to controls, the second study showing a 27 % reduction in cervical stroke volume would indicate an equal reduction in relative compliance between the spinal canal and the veins. The first study, where the spinal canal volumes were equal, would indicate the veins should be of somewhat lower relative compliance than the spinal canal. Thus, in both MS and NPH there is a reduction in spinal canal compliance.

### Venous sinus compliance

In this study, the normal young, direct 31 % of the arterial stroke volume to the sagittal sinus and 8.7 % to the straight sinus. In MS this was much less i.e. 16 % of the arterial stroke volume was directed towards the sagittal sinus and 4.4 % to the straight sinus. Given the arterial stroke volume was larger in MS than in controls, this would tend to indicate that the compliance of the cortical veins leading to the sinuses is very low and thus resist compression in MS. In NPH the percentage of the arterial pulse directed to the sinuses was not significantly different to normal, suggesting as previously noted, that the reduction in compliance is probably equally shared between the spinal canal and veins.

### A common pathophysiology of MS and NPH

There appears to be a common pathophysiological mechanism underlying MS and NPH. Both disorders generate collateral blood flow, bypassing the sagittal sinus, suggesting an increase in sinus pressure. Both disorders show a decrease in compliance compared to controls. Confounding the lower compliance in MS is the increased arterial stroke volume. Both conditions show a reduction in relative spinal canal and venous compliance but the relative changes between the spine and veins vary.

The arterial pulse pressure within the arteries of the neck represents a source of potential energy. In order for this energy to be removed from the arterial circulation before the capillary bed, it is necessary for the volume increase generated by the pulse pressure to be directed to the subarachnoid space and veins. If the compliance of the spine and veins is low, the pulse pressure damping is reduced and there is thought to be an increase in capillary pulse pressure, causing capillary disruption. In human hydrocephalus, the capillary wall shows blood–brain barrier dysfunction with increased vesicular and vacuolar transport, open inter-endothelial junctions, thin and fragmented basement membranes and discontinuous perivascular astrocytic end feet. The findings suggested an inter-endothelial route either for hydrocephalic oedema formation or resolution [49].

In addition to the capillary bed being affected, the breakdown in the windkessel mechanism may lead to an increased pulse pressure in the veins of the brain [17]. NPH is associated with a doubling in the subarachnoid space pulse pressure [41, 42] and the current data suggests an increase in subarachnoid pulse pressure may also occur in MS. The cortical veins and the vein of Galen are thin walled, so the pulse pressure within these structures will be identical to the CSF pulse pressure. Despite the suggested increase in cortical vein pulse pressure, there is a failure of the pulse pressure being converted into pulsatile flow in the veins in MS and in NPH [50]. The intracranial venous system is without valves so the pulse pressure is free to travel in both directions i.e. toward the sinuses and towards the smallest venules. In NPH there is thickening of the walls of the smallest venules which is termed perivenular collagenosis [51]. A similar process can be seen within the eyes of MS patients, which occur in the absence of demyelination (there is no myelin in the retina). Measurement of the retinal artery to retinal vein pulse delay (similar to the AVD) indicates the vessels are reduced in compliance by 25 % in MS compared to controls [52]. Reducing the compliance of the venules will make conversion of the pulse pressure to pulsatile flow ineffective. Therefore, the retinal veins will have a high pulse pressure. The retinal veins in MS are associated with a breakdown of the blood-retinal barrier with venous fluorescein leakage. Leaking venules are associated with a proteinaceous perivenous sheathing, which occurs especially at arterial/venous crossover points [53] where the venous pulse pressure would be at a maximum. All of these findings are analogous to those in NPH as already described.

If the pathophysiology between NPH and MS is so similar one might expect that there would be some overlap in presentation. The absolute compliance in NPH is much lower than MS and the intracranial pulse pressure is probably higher but there may be a few patients who present with both conditions. There have been a few isolated case reports of patients with MS who had shunt responsive hydrocephalus [54, 55]. It is likely that more patients exist but they may be misinterpreted as atrophy rather than NPH. Similarly, a theory has been put forward that syringomyelia secondary to Chiari I malformation develops in the cervical cord, due to an increase in local CSF pulse pressure secondary to reduced spinal canal compliance (pulse wave myelopathy) [56]. If this were true, then there should be a correlation between MS and syrinx formation in those whose spinal canal compliance dropped low enough. Firstly, as noted previously, the pulse volumes were significantly increased in the epidural veins in the spine in adolescents with MS [45], suggesting an increase in spinal pulse pressure and secondly, syringomyelia is noted in 4.5 % of patients with MS [57]. This suggests that the syrinx is more likely to be a consequence of the MS pathology rather than a coincidence [57].

### Limitations of the method

There is no ideal plane to measure the arterial inflow with a single MR acquisition. A single arterial acquisition was required in this study to minimise the time the patient spent in the scanner, because the flow measurements were acquired after a full 40 min diagnostic MS study. Measured in the current plane, the carotid arteries were at the skull base and the extracranial carotid segments were excluded. However, the subarachnoid extent of the vertebral vessels extends from the upper surface of C1 so the subarachnoid vertebrals and the lower basilar artery were not measured. The effect was to miss some of the blood flow supplying the cerebellum, brainstem and spinal cord and also underestimate the arterial stroke volume contributed by the expansion of the posterior fossa vessels. Effectively, the current study measured the supratentorial blood flow and pulsation. The alternative would have been to place the arterial acquisition in the neck at C2 similar to Qvarlander et al. [48]. However, although all of the arterial flow would be measured, large segments of the extracranial carotid and vertebral arteries would have been added. The vessels within the subarachnoid space are constrained by the water bath of the CSF and the compliance of the walls of the container. The extracranial vessels are not so constrained so the neck positioning will significantly overestimate the arterial stroke volume (the normal elderly in Qvarlander et al. showed an arterial stroke volume of 1.6 times the current study, despite the mean flow being not significantly different). The positioning of the venous acquisition means the venous flow and pulsation were also being measured supratentorially, similar to the arterial measurement. The neck positioning of the venous acquisition would exclude the comparison between the straight and sagittal sinuses performed by this study and also vastly overestimate the venous stroke volume. The venous stroke volume in Qvarlander et al. was 6.3 times the sagittal sinus stroke volume for the normal controls. The AVD would also have been invalid if large sections of compliant extracranial vessels were added.

The venous stroke volume and flow as measured represent only a portion of the total. The sagittal sinus and straight sinus outflow being 60 % of the arterial inflow in the young controls. Other blood flow and pulsation leaves the brain via the basal sinuses, ophthalmic veins, emissary veins and direct connections to the transverse and sigmoid sinuses. This is the reason the percentage of the arterial stroke volume directed to the spinal canal, sagittal sinus and straight sinuses can all be reduced in MS because the extra is directed to the basal sinuses. Although the relative compliances of the spinal canal and the sinuses measured by the current study were reduced (and the total compliance reduced), if the basal sinuses were unchanged in compliance then a larger percentage of the pulse volume would be directed to them, or the Monro–Kellie doctrine would be violated. There is no way to measure the basal sinus components directly except by subtraction of the known stroke volumes. The acquisition planes as used were identical to the earlier studies [16, 17] and are therefore directly comparable.

The measurement of the AVD attempts to find the centre of each pulse wave, however, due to the waves not being perfect sine waves the centre based on the timing (as currently used) may not represent the centre of the volume of blood expansion but the error is expected to be small.

## Conclusions

The pulsation pathophysiology of MS is similar to NPH suggesting that there may be an underlying pulse wave encephalopathy component to this disease which is being masked by the more obvious inflammatory overlay. It is uncertain if the neurodegenerative component is primary or secondary but the pulse wave encephalopathy which ensues could account for some of the chronic symptoms of MS and treatments aimed at altering the pulse wave propagation should be investigated.

## Additional file

10.1186/s12987-016-0041-2 Blood flow and pulsation measurements.

## Abbreviations

AVD: arteriovenous delay
AD: Alzheimer’s disease
C2: cervical vertebra 2
MS: multiple sclerosis
NPH: normal pressure hydrocephalus
NY: normal young
NE: normal elderly
SSS: superior sagittal sinus
ST: straight sinus
SD: standard deviation
